# Administration of Desloratadine for Postorgasmic Illness Syndrome in a Japanese Teenager

**DOI:** 10.1002/iju5.70128

**Published:** 2026-01-14

**Authors:** Kaoru Ito, Ko Kobayashi, Kohei Hashimoto, Wakako Yorozuya, Ko Okabe, Seisuke Nofuji, Yasuyuki Sakai, Yuki Kyoda, Toshiaki Tanaka, Noaya Masumori

**Affiliations:** ^1^ Department of Urology Sapporo Medical University School of Medicine Sapporo Japan

**Keywords:** antihistamine, ejaculatory disorder, masturbation, postorgasmic illness syndrome, wet dreams

## Abstract

**Introduction:**

Postorgasmic illness syndrome is a rare condition occurring after ejaculation. This syndrome can significantly affect the quality of life of patients.

**Case Presentation:**

A 17‐year‐old boy suffered from annoying fatigue, headache, photophobia and the sensation of a flu‐like state. He did not know the trigger of these symptoms. He visited a psychiatry clinic where he was diagnosed with depression. Although he received administration of selective serotonin reuptake inhibitors, his symptoms continued. Then he noticed that his symptoms occurred after ejaculation by masturbation or wet dreams. He visited our outpatient clinic when he was 19 years old. We administered desloratadine. One month later the general malaise after ejaculation improved. Thereafter, all symptoms after ejaculation disappeared.

**Conclusions:**

A Japanese teenager suffering from postorgasmic illness syndrome was successfully treated with desloratadine. We believe that our experience will contribute to elucidating and treating this condition in the future.

## Keynote Message

Postorgasmic illness syndrome (POIS) is a rare syndrome occurring after ejaculation. We report a case of POIS treated with desloratadine, which, to the best of our knowledge, is the first use of this treatment for POIS.

## 1 Introduction

Postorgasmic illness syndrome (POIS) is a rare syndrome that occurs after ejaculation. There are many postejaculatory symptoms; for example the sensation of a flu‐like state, extreme fatigue or exhaustion, weakness of musculature, experiences of feverishness or perspiration, mood disturbances and/or irritability, memory difficulties, concentration problems, incoherent speech, nasal congestion or a runny nose, and itchy eyes [1]. These symptoms commonly occur within a few hours after ejaculation and last between 2 and 7 days, and then disappear spontaneously. The etiology of this syndrome is unknown and there is no definitive treatment for patients with POIS, although a wide variety of treatments have been reported [1, 2]. We report a case of POIS treated with desloratadine, which to the best of our knowledge, is the first use of this treatment for POIS.

## 2 Case Presentation

A 17‐year‐old boy presented with annoying fatigue, headache, photophobia and a sensation of a flu‐like state. He did not know the trigger of these symptoms. He visited an ophthalmology clinic but no abnormalities were found; then he visited a psychiatry clinic where he was diagnosed with depression. Although he received selective serotonin reuptake inhibitors, his symptoms continued. He had to stop going to high school because of his persistent symptoms. Then he noticed that his symptoms occurred after ejaculation. Thinking back, the symptoms started after ejaculation when he was 15 years old, including by masturbation or wet dreams. They occurred within a few hours after ejaculation and disappeared spontaneously within a month. Because the symptoms continued, he visited our outpatient clinic when he was 19 years old. Although he avoided masturbation, the symptoms continuously occurred after wet dreams. There were no abnormal findings on his external genitalia or in a general blood test. His free testosterone level was 10.8 pg/mL, in the normal range.

We diagnosed his condition as POIS because his symptoms occurred after ejaculation and disappeared spontaneously. We started the administration of desloratadine as the treatment after obtaining the approval of the clinical ethics committee in our hospital (no. 23‐030) because desloratadine is not approved for the treatment of POIS in Japan. One month after beginning desloratadine, the general malaise after ejaculation improved. Thereafter, all symptoms after ejaculation disappeared. After 6 months of continued desloratadine administration, the treatment was discontinued. His symptoms did not reoccur after the cessation of the treatment.

## 3 Discussion

POIS is a rare but debilitating cluster of postejaculatory symptoms affecting men [1, 2]. In 2002 Waldinger and Schweitzer first reported two cases that exhibited flu‐like symptoms, including myalgias, fatigue, and intense warmth, and local allergic signs, including sore throat, postnasal drip, skin erythema, and a burning sensation in the eyes that occurred shortly after ejaculation [1, 2, 3]. Although much time has passed since the first report, the prevalence and incidence of POIS have remained unknown owing to the paucity of studies [1, 2]. There is a wide variety of reported treatments including antihistamines, selective serotonin reuptake inhibitors, benzodiazepines, niacin, and non‐steroidal anti‐inflammatory medications [1, 2]. Treatment efficacy varies depending on the treatment, with antihistamines reported to have a success rate of 50% [2].

We administrated desloratadine in this case. It is an antihistamine, others of which have been reported as treatments for patients with POIS‐like symptoms [1, 2]. A previous report suggested that antihistamines were the most common trialed treatments [2]. Therefore, we selected antihistamines at first. To the best of our knowledge, this is the first report of the use of desloratadine for a POIS patient. Desloratadine is one of the medications used in first‐line therapy for allergic rhinitis, which is a disease affecting a large number of patients [4]. It is also a relatively new medicine and its low adverse event rate has been reported [5]. We thought that it would be better to select a new medicine widely used for other diseases such as allergic rhinitis. Therefore, we believe that this case report provides extremely useful information regarding the treatment of a rare disease using a widely used medication.

There have been three case reports of POIS in the English literature from Japan [6, 7, 8]. Different treatment options were selected in each report. One of these reported testosterone replacement therapy for a POIS patient with hypogonadism [7]. Although the etiology of POIS is unknown, the most widely accepted hypothesis proposes that it is an immunologic phenomenon [1]. This suggests that POIS is an autoimmune or allergic disorder that generates an inflammatory reaction to a substance in seminal fluid. However, the causes of POIS may sometimes be different from allergy‐related conditions. Therefore, treatments for POIS should be selected precisely according to the pathophysiology [7].

In this case, we did not perform a prick or skin test [6]. Although one of the reasons for POIS is immunogenic pathogenesis [9], the diagnosis of POIS is based on the characteristic symptoms [1, 2]. Moreover, taking a sample of semen can be invasive for patients with POIS. Therefore, we avoided these tests in this case.

Why the symptoms resolved after desloratadine discontinuation was unclear. One speculation is that this patient may have become more tolerant of his semen while taking desloratadine. However, this is just our speculation. We believe that if there are more reports of similar outcomes in the future, we may be able to get closer to resolving this issue.

The physical and psychological effects of POIS can significantly affect the quality of life of patients [1]. In this Japanese teenager's case, he had to stop going to high school because of his persistent symptoms. It was a very unfortunate outcome caused by this rare condition, which is underdiagnosed and underreported [1, 2]. Therefore, it is not a well‐known disease in general. If POIS were well known, this Japanese teenager might have been able to notice this disease himself and receive appropriate treatment much earlier. We hope that future studies and the accumulation of cases, including our case, will contribute to elucidating and treating this disease.

## 4 Conclusions

We reported a Japanese teenaged case of POIS treated with desloratadine, which to the best of our knowledge is the first use of this treatment for this syndrome. We believe that our experience will contribute to elucidating and treating this rare disease.

## Ethics Statement

Approval of the research protocol by an Institutional Review Board and the approval number: no. 23‐030.

## Consent

Written informed consent was obtained from the patient and his mother.

## Conflicts of Interest

Naoya Masumori and Kohei Hashimoto are Editorial Board members of IJU Case Reports and co‐authors of this article. To minimize bias, they were excluded from all editorial decision‐making related to the acceptance of this article for publication.

## Data Availability

The data that support the findings of this study are available on request from the corresponding author. The data are not publicly available due to privacy or ethical restrictions.
